# Neoadjuvant Therapy in Differentiated Thyroid Cancer

**DOI:** 10.1155/2016/3743420

**Published:** 2016-09-22

**Authors:** Rajan P. Dang, Daniel McFarland, Valerie H. Le, Nadia Camille, Brett A. Miles, Marita S. Teng, Eric M. Genden, Krzysztof J. Misiukiewicz

**Affiliations:** ^1^Department of Medical Education, Icahn School of Medicine at Mount Sinai, New York, NY, USA; ^2^Department of Hematology-Oncology, Tisch Cancer Institute, Icahn School of Medicine at Mount Sinai, New York, NY, USA; ^3^Department of Otolaryngology-Head and Neck Surgery, Icahn School of Medicine at Mount Sinai, New York, NY, USA

## Abstract

*Objectives*. Invasion of differentiated thyroid cancer (DTC) into surrounding structures can lead to morbid procedures such as laryngectomy and tracheal resection. In these patients, there is a potential role for neoadjuvant therapy.* Methods*. We identified three studies involving the treatment of DTC with neoadjuvant chemotherapy: two from Slovenia and one from Japan.* Results*. These studies demonstrate that in selected situations, neoadjuvant chemotherapy can have a good response and allow for a more complete surgical resection, the treatment of DTC. Additionally, the SELECT trial shows that the targeted therapy lenvatinib is effective in the treatment of DTC and could be useful as neoadjuvant therapy for this disease due to its short time to response. Pazopanib has also demonstrated promise in phase II data.* Conclusions*. Thus, chemotherapy in the neoadjuvant setting could possibly be useful for managing advanced DTC. Additionally, some of the new tyrosine kinase inhibitors (TKIs) hold promise for use in the neoadjuvant setting in DTC.

## 1. Introduction

Neoadjuvant chemotherapy is integrated into the treatment of several cancers, including head and neck squamous cell carcinoma (hnSCC) [1]. In a phase III study by Licitra and Vermorken, rates of mandibular resection were significantly lower in hnSCC patients receiving neoadjuvant chemotherapy, thus demonstrating the ability of this treatment to preclude morbid and aggressive surgery [2]. On the other hand, neoadjuvant chemotherapy does not have an established role in differentiated thyroid cancer (DTC). Patients with thyroid cancer are historically treated with surgical resection of the primary tumor and locoregional nodal metastasis followed by radioiodine ablation (RAI).

While the majority of thyroid cancers present as locally resectable tumors with minimal surgical morbidity, occasionally the primary tumor has invaded critical structures, complicating the surgical treatment plan. Direct invasion of the larynx, trachea, pharynx, esophagus, recurrent laryngeal nerve, strap muscles, and/or carotid artery occurs in 7%–16% of patients with thyroid cancer [3]. Extensive invasion of the primary tumor into surrounding structures can necessitate relatively morbid procedures such as laryngectomy, tracheal resection, and esophageal-pharyngeal resection. Nevertheless, the importance of complete resection cannot be overstated as approximately 80% of patients who die of thyroid cancer have locoregional recurrence [4]. Certainly, a small number of patients also present with disease that may not be amenable to surgical resection, or in some cases the patient may refuse surgical resection due to the surgical morbidity. In these cases, a discussion regarding the role of neoadjuvant chemotherapy or radiotherapy is warranted. Unfortunately, neoadjuvant external beam radiation therapy (XRT) can lead to fibrosis, which can make resection of the thyroid more challenging for the surgeon. In these situations, there is a potential role for neoadjuvant chemotherapy and there are data that suggest that in selected patients, this strategy could prove to be beneficial for the treatment of extensively invasive DTC.

In this review, we will describe the potential role of neoadjuvant therapy in the treatment of DTC. Chemotherapy generally has a limited role in DTC as it is associated with minimal survival benefit when used as single modality therapy. In the neoadjuvant setting, however, the most important factor for therapy is tumor response. A favorable tumor response may offer the patient and surgeon significant improvement in the ability to provide an oncologic surgical resection. Given that data on neoadjuvant therapy in DTC is extremely limited, we will first discuss chemotherapy in its more established role in the metastatic setting in an effort to assess tumor response rates as they might pertain to neoadjuvant therapy. We will then describe the results of studies demonstrating the efficacy of neoadjuvant chemotherapy in DTC as well as the data for newer, targeted agents in an effort to identify the best possible options for neoadjuvant therapy in DTC.

## 2. Review

### 2.1. Chemotherapy in Metastatic DTC

Although a limited number of chemotherapies have been used effectively in the neoadjuvant setting, chemotherapy has not been shown to be particularly effective in the metastatic setting. The only FDA-approved chemotherapy for DTC is doxorubicin, which offers minimal benefit and is associated with toxicity for patients with metastatic DTC. Doxorubicin was initially introduced to clinical practice due to a study by Gottlieb et al., which described 6 case reports of patients with DTC treated with various chemotherapy agents given alone or in combination [5]. Two patients (33.3%), both of whom were treated with doxorubicin, had a partial response (PR). These results led to a prospective clinical trial studying 15 patients with DTC treated with doxorubicin, in which a 33% PR was observed [6]. See Table 1 for details [5–10]. However, multiple, larger, and better-designed trials over the past 30 years have demonstrated that doxorubicin may not be as effective as previously believed [31]. For example, Matuszczyk et al. conducted a retrospective study in which doxorubicin was given to patients with DTC, finding only a 5% PR [7].

Given the limited efficacy of doxorubicin as monotherapy, other chemotherapy combinations have been evaluated in clinical trials, but also with disappointing results. Williams et al. described a study with 22 patients with advanced thyroid cancer of all histological subtypes treated with doxorubicin and cisplatin [8]. They observed a 9.1% PR and the treatment was associated with considerable toxicity, including one treatment-related death. Another study by Shimaoka et al. described patients that were randomized to doxorubicin with cisplatin or doxorubicin alone [9]. Among a group of 35 patients with DTC, the overall response rate (ORR) for combination therapy (16%) was inferior to that of monotherapy (31%).

While these results for both doxorubicin monotherapy and combination chemotherapy are discouraging for the treatment of metastatic DTC this data should be interpreted with caution. Chemotherapy is typically reserved for RAI-refractory cases of DTC, and thus the biology involved in these cases may be different from that seen in the preoperative setting [31, 32].

The following studies, on the other hand, explore the efficacy of doxorubicin as well as various other agents given as neoadjuvant chemotherapy in DTC.

### 2.2. Neoadjuvant Chemotherapy in DTC

We identified three studies involving the treatment of DTC with neoadjuvant chemotherapy: two from Slovenia and one from Japan. Response rates for these three studies are summarized in Table 2 [11–13]. Rates of residual tumor after resection are summarized in Table 3 [11, 12, 14].

One of the studies from Slovenia described a retrospective, nonrandomized study of 29 patients with T3 or T4 follicular or Hurthle cell thyroid cancers treated from 1979 to 2004 in which the tumor was considered inoperable [11]. Mean age was 60.83 years. Mean tumor diameter was 9.3 cm and extrathyroid growth was seen in 15/29 patients (51.7%). Regional metastases were present in 6 patients (20.7%) and distant metastases in 12 (41.4%). Chemotherapy consisted of vinblastine for 19 patients (65.5%), vinblastine with doxorubicin for 5 patients (17.2%), and other regimens for the remaining 5 patients (17.2%). Four patients (13.8%) were also treated with preoperative XRT. Surgery was performed when the tumor was reduced after chemotherapy and/or XRT and the surgeon judged the tumor resectable. The median interval between the beginning of chemotherapy and surgical procedure was 36 days (range: 4–173 days). Tumor size was decreased by >50% in 13 patients (44.8%). For patients with distant metastases, tumor size decreased by >50% in only 17% of patients, while in patients without distant metastases, tumor size decreased by >50% in 65% of patients. Histopathology revealed wide areas of tumor necrosis in 7 patients (24%). Tumor resection after chemotherapy was performed in all patients, resulting in R0 (defined as with no residual tumor), R1 (microscopic residual tumor), and R2 (macroscopic residual tumor) resections in 15 (51.7%), 10 (34.5%), and 4 (13.8%) cases, respectively. Total thyroidectomy was performed in 24 patients (82.6%) and lobectomy was performed in 5 patients (17.2%). Toxicity data was not reported.

Similarly, the same group in Slovenia described a retrospective, nonrandomized study of 16 patients with T3 or T4 papillary thyroid cancer treated from 1988 to 2005 in which the tumor was considered to be inoperable [12]. Mean age was 63.06 years. Mean tumor diameter was 9.7 cm and extrathyroid growth was present in 13 patients (81.3%). Regional metastases were present in 10 patients (62.5%) and distant metastases in 7 (43.8%). Chemotherapy consisted of vinblastine in 11 cases (68.8%), vinblastine with doxorubicin in 2 cases (12.5%), and other regimens in 3 cases (18.8%). Four patients were treated with preoperative XRT (25%). Surgery was performed when the tumor was reduced after chemotherapy and/or XRT and the surgeon judged the tumor resectable. The median interval between the beginning of chemotherapy and surgical procedure was 28 days (range: 7–161 days). After chemotherapy, tumor size decreased by >50% in 7 patients (44%). R0, R1, and R2 resection was performed in 2 (12.5%), 10 (62.5%), and 4 (25%) cases, respectively. Total thyroidectomy was performed in 11 patients (68.8%) and lobectomy in 5 patients (31.3%). No toxicity was reported. Interestingly, these two studies from Slovenia describe very different rates of residual tumor after resection (see Table 3). Given that response rates were similar between the first and second study, average tumor size was similar, and the surgeries were performed at the same institution over a similar period of time; this difference is difficult to account for. It is likely due to a higher rate of extrathyroid growth in the second study (81.3%) when compared to that of the first study (51.7%). Given this difference in extrathyroid growth and resections between the two groups, it is possible that papillary thyroid carcinoma can be more invasive and consequently more difficult for the surgeon to resect completely. These results are also difficult to characterize given the variety of chemotherapy strategies utilized and the time period over which the reviews were performed.

An additional study from Japan evaluated the effect of weekly paclitaxel chemotherapy in 3 patients with papillary thyroid cancer with a squamous cell carcinoma component (a very aggressive tumor with behavior resembling anaplastic thyroid cancer) [13]. Weekly chemotherapy was performed as induction for 2 of the patients, and all patients underwent locally curative surgery with weekly adjuvant chemotherapy after surgery. The response to chemotherapy was evaluated based on RECIST 1.1 criteria. The first patient was a 70-year-old woman with a solitary 5.9 cm thyroid tumor and multiple node metastases. She additionally had an incomplete right laryngeal nerve paralysis, likely due to tumor invasion. CT scan revealed multiple lung metastases. She received weekly induction paclitaxel at 80 mg/m^2^ for 3 cycles. The tumor size decreased by 45%, and lung metastases disappeared after induction. The patient underwent total thyroidectomy with modified radical neck dissection, and the tumor was completely resected. Lung metastases were identified again 6 months after surgery. The patient received 13 cycles of weekly paclitaxel, but the metastases enlarged gradually. The patient died 21 months after diagnosis and 18 months after surgery. The second patient was a 68-year-old woman with a 4.4 cm thyroid tumor and a single cervical node metastasis, without distant metastases. The patient received induction therapy with weekly paclitaxel (again at 80 mg/m^2^) for 2 cycles. The tumor size decreased by 15%; this result was evaluated as stable disease (SD). The patient underwent total thyroidectomy and modified neck dissection, and the tumor was completely resected. At the time of publication of this study, the patient was alive 29 months after her diagnosis and 27 months after surgery with no evidence of recurrence. No toxicity was reported for either patient receiving induction therapy.

The results from these studies indicate that neoadjuvant chemotherapy may have some effectiveness in DTC and should be considered in select cases. Neoadjuvant regimens consisted mostly of vinblastine, doxorubicin, and/or paclitaxel and offered superior responses compared to doxorubicin alone (or with cisplatin). Of note, patients in these studies were typically older, with mean ages over 60. Thus, neoadjuvant chemotherapy can be both effective and tolerable in this population.

Despite these results, the data is limited to these two retrospective studies and two patients from a case series. Interestingly, none of these studies reported toxicity associated with neoadjuvant chemotherapy. While it is possible that neoadjuvant chemotherapy was well tolerated by the patients in these studies, the lack of concrete toxicity data is a significant weakness. Ideally, recommendations for treatment using neoadjuvant therapy would be based on randomized controlled trials (RCTs); however it is unlikely that enough patients could be accrued to power such a study. Consequently, there is a need for more retrospective studies and case reports in order to enhance the current data for this treatment.

### 2.3. Targeted Agents

The studies described thus far in this review have all used cytotoxic chemotherapy, which is relatively indiscriminate in its toxicity. In contrast, newer targeted therapies are based on specific genetic properties of tumors and are generally less toxic as a result. In particular, tyrosine kinase inhibitors (TKIs) have shown promise in the treatment of DTC. In 2013, sorafenib was approved for the treatment of metastatic DTC. Furthermore, lenvatinib was found to extend progression-free survival (PFS) in RAI-resistant DTC in the phase III SELECT trial and was approved for the treatment of metastatic DTC in 2015. Although there are no adjuvant data for these drugs, the phase III trial data is thought provoking for the potential role of targeted therapy in the neoadjuvant setting.

The double-blind, randomized, multicenter, phase III DECISION trial examined sorafenib efficacy and safety versus placebo in patients with progressive RAI-refractory DTC [15]. A total of 417 patients were randomized; 207 were to sorafenib and 210 to placebo. Ninety-six percent of patients had metastatic disease. ORR in the sorafenib versus placebo arms was 12.2% and 0.5% (all responses were partial responses). Stable disease ≥6 months was achieved in 42% versus 33% for sorafenib versus placebo, respectively. The most common any-grade treatment-emergent adverse events in the sorafenib arm were hand-foot skin reaction, diarrhea, alopecia, rash/desquamation, fatigue, weight loss, and hypertension. Tolerability in this study was consistent with the known sorafenib safety profile. Consequently, this study demonstrated a significant advantage of sorafenib over placebo with limited toxicity. However, the 12.2% ORR is still very low and is not ideal for neoadjuvant therapy. This result is consistent with other phase II data regarding sorafenib [33]. See Table 4 for a summary of studies describing targeted therapy in DTC [15–26].

The multicenter, randomized, placebo-controlled, phase III SELECT trial presented at the American Society of Clinical Oncology (ASCO) 2014 annual meeting examined the efficacy and safety of lenvatinib versus placebo in patients with RAI-refractory DTC [24]. Patients were allowed to have received ≤1 prior vascular endothelial growth factor receptor- (VEGFR-) targeted therapy. A total of 392 patients were randomized in a 2 : 1 ratio to lenvatinib or placebo. Complete response (CR) rates were 1.5% for lenvatinib and 0% for placebo, and PR rates were 63.2% for lenvatinib and 1.5% for placebo. Median time to response was 2.0 months. The most common lenvatinib treatment-related adverse events were hypertension (68%), diarrhea (59%), decreased appetite (50%), weight loss (46%), and nausea (41%). Lenvatinib-related grade ≥3 adverse events were hypertension (42%), proteinuria (10%), weight loss (10%), diarrhea (8%), and decreased appetite (5%). The dose was reduced in 78.5% of patients and discontinued due to adverse events in 14.2% of patients. Consequently, response rates for lenvatinib were far superior to those of sorafenib. Additionally, these results show that lenvatinib is a good candidate for use as neoadjuvant therapy in DTC, given its short time to response and a high response rate.

A phase II trial conducted by Bible et al. describes pazopanib as a promising new targeted agent for DTC [26]. Thirty-seven patients with metastatic, rapidly progressive, RAI-refractory DTC received pazopanib until disease progression and/or drug intolerance. Up to two previous therapies were allowed. Confirmed partial responses were seen in 18 patients (49%). Sixteen patients (43%) required dose reductions due to adverse events, the most common (of any grade) being fatigue (78.4%), skin and hair hypopigmentation (75.7%), diarrhea (73%), and nausea (73%). Two patients died during treatment, though they each had preexisting contributory disorders.

These data demonstrate that lenvatinib and pazopanib are promising agents for neoadjuvant therapy in DTC. The former has a documented short time to response, and both had good response rates with low toxicity. Furthermore, given that these drugs would be given for a short time period if administered as neoadjuvant therapy, long-term toxicity would also be minimal. However, there exists the theoretical concern of selecting for tumor cell clonality resistant to these new therapies if they are used early in the course of treatment as neoadjuvant therapy. If such a patient were to develop RAI-resistant metastases, the treatment options for the patient would indeed be poor. However, this scenario is very unlikely, and its possibility should not preclude the consideration of targeted agents as neoadjuvant therapy in patients that could benefit from this treatment.

Sorafenib, on the other hand, had a relatively low response rate of 12% in a phase III trial, which is consistent with data from prior phase II trials. The different biochemical properties of these drugs may contribute to the considerable differences in response rates between sorafenib and lenvatinib/pazopanib (see Table 5 [27–30] for a summary of the molecular targets of these TKIs). Additional studies could be helpful in elucidating these differences. While ineffective on its own, there is the possibility that sorafenib could be effective in conjunction with other targeted therapies or cytotoxic chemotherapies. Further studies could elaborate on the potential of combining different targeted therapies with each other or with cytotoxic chemotherapy.

## 3. Discussion

Neoadjuvant chemotherapy can preclude the need for aggressive and morbid surgery, such as laryngectomy, tracheal resection, or pharyngoesophageal resection [3]. It can also render an inoperable tumor operable and can improve a patient's prognosis by allowing a more complete resection of a patient's tumor. Furthermore, it can act on metastatic disease if present and add additional value to postoperative RAI, which is generally more efficacious for patients with only remaining microscopic disease rather than macroscopic.

The current literature describes R0 and R1 resections as offering improved survival when compared to R2 resection [34–40]. However, the respective effects of R0 and R1 resections on prognosis are more controversial. The clinical benefits of complete resection are apparent in a retrospective single-center study by Hartl et al., in which they described 46 patients with DTC invading the trachea, larynx, pharynx, or esophagus, none of whom received neoadjuvant chemotherapy [14]. R0, R1, and R2 surgeries were performed in 22 (49%), 24 (51%), and 0 (0%) patients, respectively. All patients received postoperative RAI, and 23 (50%) received adjuvant XRT. The 10-year local control rate was 100% for R0 patients and 75% for R1 patients; 5-year disease-specific survival was 95% for R0 patients and 84% for R1 patients.

Of note, the study by Hartl et al. seems to describe a more favorable R0/R1/R2 than the two Slovenian studies, despite the lack of neoadjuvant therapy in the former study. However, the tumors described in the Slovenian studies were considered inoperable at the start and consequently were likely larger and more extensive from the beginning than those described in the study by Hartl et al. Also of note, the studies from Slovenia seem to demonstrate that neoadjuvant therapy is more effective in patients without metastases. This is potentially due to the less aggressive biology of tumors that have not yet metastasized.

Neoadjuvant therapy could also be useful in certain specialized cases, for instance, those involving deep tracheal invasion or PET-positivity. For instance, some studies demonstrate that tracheal shaving is an effective procedure for the treatment of tracheal invasion when compared to sleeve resection (a more aggressive but more definitive procedure) [41, 42]. However, in cases of deep invasion, tracheal shaving has a greater risk of leaving gross disease behind [11]. Thus, neoadjuvant therapy could potentially reduce the extent of invasion and reduce the risk of residual disease after surgical resection. Likewise, neoadjuvant therapy may be useful in PET-positive DTC tumors. Prior studies have demonstrated that PET-positive tumors are often aggressive and RAI-resistant [43, 44]. In these situations, neoadjuvant therapy might be a valuable tool in a setting in which treatment options are limited.

While VEGF is critical for tumor angiogenesis and growth, it is also imperative to wound healing [45]. Thus, a significant possible drawback of the use of anti-VEGF TKIs, such as sorafenib, lenvatinib, or pazopanib, as neoadjuvant therapy is the possibility of impaired wound healing after surgery. Consequently, the use of these agents as neoadjuvant therapy would potentially require a waiting period between drug administration and surgery, possibly allowing the tumor time to regrow. That being said, the current literature contains few reports of impaired healing or wound dehiscence following therapy with anti-VEGF TKIs. For instance, in the Phase III SELECT trial of lenvatinib, 6 of 118 deaths were determined to be treatment-related, and none of these were related to surgical or wound problems [46]. Of note, the package insert for lenvatinib reports a 0.8% rate of impaired healing and 0.4% of wound dehiscence [47]. Antiangiogenic therapy could also potentially lead to fistula formation in these patients given the potential for radiation and/or surgery around the trachea and esophagus. Tracheoesophageal fistula has been reported in lung cancer patients after treatment with the VEGF inhibitor bevacizumab in addition to radiation [48–50]. In another study, 5 of 43 head and neck cancer patients treated with bevacizumab and chemoradiotherapy developed an aerodigestive fistula [51]. However, a 2014 literature review conducted by Blevins et al. only yielded one case of antiangiogenic therapy for thyroid cancer resulting in aerodigestive fistula, though details regarding the patient's history were not published [52]. In the same paper, Blevins et al. report two cases of aerodigestive fistula in patients with DTC treated with antiangiogenic TKIs [52]. Both of these patients were previously treated with surgery and radiation. Thus, these data demonstrate a rare risk of fistula formation in patients with DTC treated with antiangiogenic TKIs.

Given that there is no data regarding the use of antiangiogenic TKIs as neoadjuvant therapy in DTC, it is difficult to speculate as to the possibility of postoperative wound dehiscence or fistula formation. Furthermore, there are no current guidelines for these drugs on how long to wait before surgery. While we believe that, in specific circumstances, neoadjuvant therapy might be a valuable tool in the treatment of DTC, these risks should be deliberated in any treatment plan. The possibility of a waiting period between drug administration and surgery should be considered while considering the risk of tumor regrowth. Given the lack of guidelines or data, these decisions should be made on the basis of the individual patient and tumor. More data need to be collected regarding the possibility of impaired wound healing or fistula formation after treatment with antiangiogenic TKIs in DTC before any recommendations on a waiting period between drug administration and surgery are made. In the opinion of the authors of this paper, an antiangiogenic TKI could be administered for approximately four months (with response first assessed after two months), and surgery could take place 4–6 weeks after stopping the drug. Again, however, additional data is necessary before establishing any guidelines for duration of therapy and the waiting period.

## 4. Conclusions and Future Directions

Currently, the role for neoadjuvant chemotherapy in DTC is not well established. However, our review shows that in selected situations neoadjuvant chemotherapy can be beneficial in the treatment of DTC. Moreover, the SELECT trial shows that the targeted therapy lenvatinib is effective and safe in the treatment of DTC and could potentially be useful as neoadjuvant therapy for this disease given its high response rate and short time to effect. Pazopanib has also demonstrated promise in phase II data, and sorafenib, while relatively ineffective alone, could possibly be useful in conjunction with other therapies.

There are still many unanswered questions regarding neoadjuvant therapy in DTC. There is still limited data on the respective effects of R0, R1, and R2 on prognosis. There are also no clear indications for the use of neoadjuvant therapy in DTC, and this decision would likely be made by the surgeon based on the extent of disease and complexity of the operation for the surgeon. It is unlikely that a sufficiently powered RCT will be conducted for this treatment, and, consequently, treatment recommendations will have to rely on strong retrospective data and case reports and series. Collaboration and cooperative research groups could possibly yield stronger data. Future studies should potentially include combinations of targeted therapies with each other or other cytotoxic chemotherapies to further elucidate the possibilities for neoadjuvant therapy in DTC.

## Figures and Tables

**Table 1:** Select studies of chemotherapy in metastatic differentiated thyroid cancer.

Author and year	Sample size	Intervention	Response
Gottlieb et al., 1972 [5]	6 with DTC.	Various single and combination agents, including doxorubicin.	33.3% PR.
Gottlieb and Hill, 1974 [6]	15 with DTC.	Doxorubicin at 45, 60, or 75 mg/m^2^ IV.	33.3% PR.
Matuszczyk et al., 2008 [7]	22 with DTC.	Doxorubicin at 15 mg/m^2^ IV weekly or 60 mg/m^2^ IV every 3 weeks.	5% PR, 42% SD, 53% PD.^*∗*^
Williams et al., 1986 [8]	22 with advanced thyroid cancer of all histological subtypes.	Doxorubicin 60 mg/m^2^ plus cisplatin 60 mg/m^2^.	9.1% PR.
Shimaoka et al., 1985 [9]	35 with DTC.	Doxorubicin 60 mg/m^2^ with cisplatin 40 mg/m^2^ or doxorubicin alone.	16% and 31% ORR for combination and monotherapy, respectively.
Matuszczyk et al., 2010 [10]	7 with DTC.	Paclitaxel 90–100 mg/m^2^ and gemcitabine 1000 mg/m^2^.	No responses observed.

Definitions: DTC: differentiated thyroid cancer, MTC: medullary thyroid cancer, ORR: overall response rate, PD: progressive disease, PR: partial response, and SD: stable disease.

^*∗*^Results for both doses (15 mg/m^2^ IV weekly or 60 mg/m^2^ IV every 3 weeks).

**Table 2:** Responses to neoadjuvant chemotherapy in differentiated thyroid cancer.

Author and year	Sample size	Intervention	Response
Besic et al., 2012 [11]	29 with T3 or T4 follicular or Hurthle cell thyroid cancer.	Vinblastine, vinblastine with doxorubicin, or other regimens.	RR 44.8%.
Besic et al., 2013 [12]	16 with T3 or T4 papillary thyroid cancer.	Vinblastine, vinblastine with doxorubicin, or other regimens.	RR 40%.
Ito et al., 2012 [13]	2 with papillary thyroid cancer with a squamous cell carcinoma component.	Paclitaxel 80 mg/m^2^.	50% PR, 50% SD.

Definitions: PR: partial response, RR: response rate, defined as decrease in tumor size by >50%, and SD: stable disease.

**Table 3:** Rates of residual tumor after resection in differentiated thyroid cancer.

Author and year	Sample size	Neoadjuvant chemotherapy	R0, R1, R2
Besic et al., 2012 [11]	29 with T3 or T4 follicular or Hurthle cell thyroid cancer.^*∗*^	Vinblastine, vinblastine with doxorubicin, or other regimens.	51.7%, 34.5%, and 13.8%.
Besic et al., 2013 [12]	16 with T3 or T4 papillary thyroid cancer.^*∗*^	Vinblastine, vinblastine with doxorubicin, or other regimens.	12.5%, 62.5%, and 25%.
Hartl et al., 2014 [14]	46 with extensively invasive DTC.	None.	49%, 51%, and 0%.

Definitions: DTC: differentiated thyroid cancer, R0: resection with no residual tumor, R1: resection with microscopic residual tumor, and R2: resection with macroscopic residual tumor.

*∗* is considered inoperable prior to neoadjuvant treatment.

**Table 4:** Summary of studies of targeted therapy in differentiated thyroid cancer.

Author and year	Sample size	Intervention	Response
Brose et al., 2014 [15] Phase III DECISION trial	417 with RAI-refractory DTC.	Sorafenib 400 mg 2x daily.	ORR 12.2% and 0.5%, SD 42% and 33%.
Marotta et al., 2013 [16]	17 with RAI-refractory DTC.	Sorafenib 400 mg 2x daily.	30% PR, 41% SD.
de la Fouchardiere et al., 2013 [17]	45 with RAI-refractory DTC.	Sorafenib.	29% PR.
Chen et al., 2011 [18]	9 with RAI-refractory DTC.	Sorafenib 200 mg 2x daily.	33% PR, 44% SD.
Cabanillas et al., 2010 [19]	13 with RAI-refractory DTC.	Sorafenib 400 mg.	20% PR, 60% SD, 20% PD.
Sherman et al., 2013 [20]	19 with DTC.	Sorafenib 400 mg 2x daily and everolimus 5 mg daily.	Papillary, 50% PR and 38% SD. Hurthle cell, 67% PR and 33% SD. Follicular, 50% PR and 50% SD.
Sherman et al., 2012 [21]	27 with recurrent/refractory DTC, 6 of whom with prior sorafenib.	Sorafenib 200 mg 2x daily with temsirolimus 25 mg weekly.	38% PR if no prior sorafenib.
Hong et al., 2011 [22]	16 with metastatic DTC.	Sorafenib 400 mg daily with tipifarnib 100 mg 2x daily.	4.5% PR and 36% SD.
Cabanillas et al., 2010 [23]	22 with metastatic DTC.	Sorafenib with tipifarnib, dose escalation trial.	7% PR, 86% SD, 7% PD.
Schlumberger, 2014 [24] Phase III SELECT trial	392 with RAI-refractory DTC.	Lenvatinib 24 mg daily.	CR 1.5% and 0%. PR 63.2% and 1.5%.
Sherman et al., 2011 [25]	58 with RAI-refractory DTC.	Lenvatinib 24 mg daily.	PR 50%.
Bible et al., 2010 [26]	37 with RAI-refractory DTC.	Pazopanib 800 mg daily.	49% PR.

Definitions: CR: complete response, DTC: differentiated thyroid cancer, ORR: overall response rate, PD: progressive disease, PR partial response, RAI: radioactive iodine, and SD: stable disease.

**Table 5:** Molecular targets of the TKIs sorafenib, lenvatinib, and pazopanib.

Drug	Targets
Sorafenib	Raf kinase, VEGFR1–3, PDGFR*β*, RET [27].
Lenvatinib	VEGFR1–3, FGFR1–4, RET, c-Kit, PDGFR*β* [28].
Pazopanib	VEGFRs, PDGFR, c-Kit [29, 30].

Definitions: FGFR: fibroblast growth factor receptor, PDGFR: platelet derived growth factor receptor, TKI: tyrosine kinase inhibitor, and VEGFR: vascular endothelial growth factor receptor.
